# Biodegradable Polymers for Gene Delivery

**DOI:** 10.3390/molecules24203744

**Published:** 2019-10-17

**Authors:** T. J. Thomas, Heidar-Ali Tajmir-Riahi, C. K. S. Pillai

**Affiliations:** 1 Department of Medicine, Rutgers Robert Wood Johnson Medical School, KTL N102, 675 Hoes Lane, Piscataway, NJ 08854, USA; 2 Rutgers Cancer Institute of New Jersey, New Brunswick, NJ 08901, USA; tajmirri@uqtr.ca; 3 Department of Chemistry-Biochemistry-Physics, University of Québec in Trois-Rivières, C. P. 500, Trois-Rivières, QC G9A 5H7, Canada; ckspillai@gmail.com

**Keywords:** gene delivery, biodegradable polymers, DNA condensation, DNA nanoparticles, polyethyleneimine, poly-L-lysine, chitosan, pullulan, dextran, hyaluronic acid, gene delivery mechanisms

## Abstract

The cellular transport process of DNA is hampered by cell membrane barriers, and hence, a delivery vehicle is essential for realizing the potential benefits of gene therapy to combat a variety of genetic diseases. Virus-based vehicles are effective, although immunogenicity, toxicity and cancer formation are among the major limitations of this approach. Cationic polymers, such as polyethyleneimine are capable of condensing DNA to nanoparticles and facilitate gene delivery. Lack of biodegradation of polymeric gene delivery vehicles poses significant toxicity because of the accumulation of polymers in the tissue. Many attempts have been made to develop biodegradable polymers for gene delivery by modifying existing polymers and/or using natural biodegradable polymers. This review summarizes mechanistic aspects of gene delivery and the development of biodegradable polymers for gene delivery.

## 1. Introduction

Gene therapy approaches are under development to treat diseases that arise from genetic abnormalities [1,2,3,4,5]. Successful gene therapy requires the efficient delivery of genetic material through the cell membrane into target sites in order to treat these diseases. Transport of DNA through the cell membrane is an inefficient process, and the mechanism(s) by which this process occurs is not clear [6,7,8,9,10,11,12]. There are two types of delivery vehicles used in gene therapy, viral and non-viral vectors, both of which present specific advantages and disadvantages [13,14,15,16]. Currently employed viral vectors include retroviruses, adenoviruses, and adeno-associated viruses, and each one of them has its own unique advantages [17,18,19,20,21]. Viral vectors are highly effective in achieving high efficiency for both gene delivery and expression, and exhibit stable long-term expression of a foreign gene when the recombinant DNA is integrated into the chromosomal DNA [22,23,24]. Major limitations of virally mediated gene delivery include limited DNA carrying capacity, toxicity, potential replication, immunogenicity, cancer formation and high cost. Non-viral gene delivery vehicles are being developed to overcome the deficiencies of viral vectors [25,26,27,28,29,30].

The concept of non-viral gene transfer can be described simply as the use of carriers other than virus which: (a) Mimics viral infection processes; (b) condenses DNA; (c) protects DNA from degradation; (d) promote cellular uptake and nuclear delivery; and (e) are non-immunogenic and non-cytotoxic [31,32]. Lipids, polymers and peptides are some of the non-viral vectors that have been developed in the past three decades [33,34,35,36,37]. These polymers interact with nucleic acids and form nanoparticles to facilitate gene delivery, with the advantages of low toxicity, cost-effectiveness, ease of production, and versatility for different applications [38,39,40,41,42,43,44]. Amongst the various non-viral vectors developed, cationic polymers have been considered as the most promising candidates with enormous potentials and advantages in comparison to their counterparts owing to their unique characteristics of forming polyelectrolyte complexes with genes and the ability to protect DNA from various enzymes [45,46]. The polymer vectors used in gene delivery should meet several requirements, including: (i) The ability to protect the genetic material from enzymatic degradation, (ii) provide long lifetime in the blood circulation, (iii) direct the genetic material to specific cellular/tissue sites, (iv) degrade and eliminate from the human body without exerting undesirable side effects, (v) the ability to enter the target cell, crossing the cell membrane and transiting through the cytosol and/or crossing the nuclear membrane to release the genetic material at the desired point of action [47].

Both synthetic and natural polymers have been used for gene delivery, and several reviews are available in the literature [48,49,50,51,52]. However, these reviews were published approximately a decade ago, and hence, we prepared this review to outline earlier research in this area and to describe developments during the past decade, especially in the area of biodegradable polymers for gene delivery applications.

## 2. DNA Condensation to Nanoparticles for Gene Delivery

**Figure 1:**
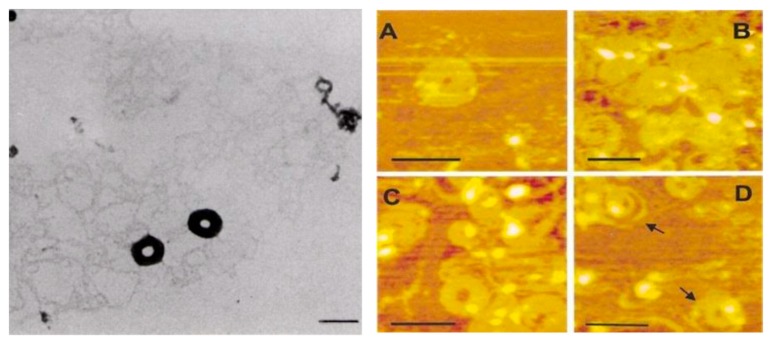
Electron micrograph of DNA treated with 200 μM of spermidine (Left). Scale bar is 100 nm. Scanning force microscopy images showing the toroid structures of pGL3 plasmid DNA formed by incubation with polyamines (Right). (**A**) 25 μM spermine; (**B**) 5 μM of pentamine (3-3-3-3, H_2_N(CH_2_)_3_NH(CH_2_)_3_NH(CH_2_)_3_NH(CH_2_)_3_NH_2_); (**C**) 2 μM of hexamine (3-4-3-4-3, H_2_N(CH_2_)_3_NH(CH_2_)_4_NH(CH_2_)_3_NH(CH_2_)_4_NH_2_(CH_2_)_3_NH_2_). The numbering system in these synthetic polyamines is the number of –CH_2_- groups between amino and imino groups. (**D**) Scale bar is 200 nm. Adapted with permission from Reference [41].

An essential requirement of DNA entry to cells is the condensation of DNA, a process by which long DNA chains are collapsed into nanoparticles of 50–200 nm diameter [8,9,26,27,40,41,42,53,54,55,56,57,58]. The interaction of multivalent cationic ligands with DNA results in the condensation of DNA to nanoparticles and facilitates DNA transport through the cell membrane [9,26]. An example of such nanoparticle formation with the natural polyamines, spermidine (H_2_N(CH_2_)_3_NH(CH_2_)_4_NH_2_) and spermine (H_2_N(CH_2_)_3_NH(CH_2_)_4_NH(CH_2_)_3_NH_2_), and synthetic polyamines are illustrated in Figure 1.

The natural polyamines have been extensively studied for their ability to collapse DNA to nanoparticles [59,60,61,62,63]. The energetic force governing the condensation of DNA is the differential between attractive and repulsive forces between DNA strands. By combining single-molecule magnetic tweezers and osmotic stress on DNA assemblies, Todd et al. separated attractive and repulsive components of the total intermolecular interaction between multivalent cation condensed DNA strands [64]. Based on measurements of different cations, including cobalt hexamine (Co(NH_3_)_6_^3+^), natural polyamines, synthetic pentamine and hexamine, Todd et al. identified two invariant properties of multivalent cation-mediated DNA interactions: Repulsive forces decay exponentially with a 2.3 ± 0.1 Å characteristic decay length, and the attractive component of the free energy was always 2.3 ± 0.2 times larger than the repulsive component of the free energy at force-balance equilibrium, irrespective of the nature of the cation [64]. The experimental measurements indicated the importance of electrostatic interactions, consistent with theories for Debye-Hückel interactions between helical line charges and with the order-parameter formalism for hydration forces [26,60]. However, ionic, structural and temperature effects were evident in the interaction of polyamines with DNA [40,41,42,65,66,67,68].

Thomas et al. tested the ability of a series of polyamine analogues for transporting a triplex DNA forming oligonucleotide in MCF-7 breast cancer cells and found that hexamine analogues of spermine were excellent candidates for oligonucleotide delivery [69]. Structurally modified oligoamines were also developed as nanocarrier gene delivery agents [70]. Polyamine-based delivery vehicles interfered with the polyamine metabolic pathway and depleted the level of natural polyamines [59,69,70]. DNA condensation by amino acids and protamine was considered to be a model for DNA packaging in the sperm [26,71]. Arginine rich polyplexes were also studied for gene delivery [72].

## 3. Mechanistic Aspects of Gene Delivery by Polymeric Vehicles

**Figure 2:**
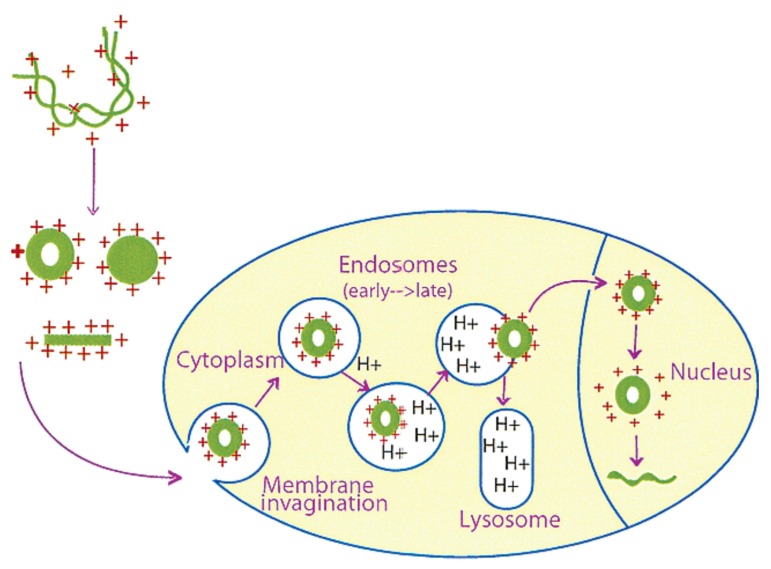
Schematic representation of DNA uptake by mammalian cells. DNA is compacted in the presence of polycations into ordered structures, such as toroids, rods, and spheroids. These particles interact with the anionic proteoglycans at the cell surface and are transported by endocytosis. The cationic agents accumulate in the acidic vesicles, increase the pH of the endosomes, and inhibit the degradation of DNA by lysosomal enzymes. They also sustain a proton influx, which destabilizes the endosome, and release DNA. The DNA then is translocated to the nucleus either through the nuclear pore or with the aid of nuclear localization signals, and decondenses after separation from the cationic delivery vehicle. Adapted with permission from Reference [9].

The cell membrane is a major barrier for the specific and efficient delivery of the nucleic acid cargo by non-viral delivery vehicles. Due to their large size and anionic nature, DNA and siRNA cannot diffuse across the cell membrane and require active internalization by endocytosis [9,26,27,73,74]. Supramolecular chemistry aids in the organization of large DNA molecules to highly organized nanoparticles of approximately 50–200 nm diameter toroids or spheres by electrostatic and/or other forms of interactions with nanocarriers, such as cationic polymers [9,26,40]. The DNA nanoparticles appear to be in a liquid crystalline state, with charge inversion conferring positive charges on their surface, and facilitating endocytosis through negatively charged proteoglycans on the cell surface [61,66,75,76]. A schematic model for the cellular uptake mechanism is given in Figure 2.

The endocytic pathway is mainly divided into four different types: (i) Phagocytosis, (ii) clathrin- mediated endocytosis, (iii) caveolae-mediated endocytosis, and (iv) macro- and micro-pinocytosis [9,12,56,77]. However, other types of processes, such as flotillin-dependent endocytosis, circular dorsal ruffles, and etosis have also been described in the literature [12,77,78]. Phagocytosis is predominant in macrophages, monocytes, neutrophils and dendritic cells and particles with a diameter of 2–3 μm diameter. The clathrin dependent endocytosis pathway is a receptor-dependent pathway, mediated by clathrin and requires GTPase dynamin. The assembly of clathrins occurs in the polyhedral lattice on the cytosolic surface of the cell membrane, which helps to deform the membrane into a coated pit with 100–150 nm size for invagination of the cargo containing the genetic material [79]. Caveolin-mediated endocytosis is a type of cholesterol and dynamin-dependent, and receptor-mediated pathway, involving 50–100 nm invaginations of the plasma membrane [80]. In contrast to the receptor-mediated pathways, macropinocytosis is a form of bulk fluid intake, mediated by cytoskeletal rearrangement to create membrane extensions to trap a large vesicle (0.2–5 μm) and fuse it back to the membrane for endocytotic uptake. It is a form of nonspecific bulk fluid uptake in the absence of any specific receptor [81]. Micropinocytosis involves the internalization of nanoparticles of <0.2 μm via both clathrin-coated and uncoated vesicles [82].

The cellular uptake pathway of DNA complexed nanocarriers can be affected by several factors, including size, surface charge, particle shape, surface modifications, cell type and culture Conditions [9,12,77]. As mentioned above, large particles are transported by phagocytosis, whereas smaller particles are taken up by micropinocytosis. In general, nanoparticles formed from non-viral gene delivery vehicles are heterogeneous in size, shape and surface charge, and hence, different types of uptake mechanisms might be active with the same type of delivery vehicle and a single cell type [83]. The cell membrane consists of anionic membrane proteins, and hence, positively charged nanoparticles are more easily transported through the cell membrane than that of negatively charged particles. However, the high positive charge will cause cytotoxicity. Therefore, moderately charged particles are ideal for cellular uptake. Zeta potential measurement is used to determine the surface charge of nanoparticles [84].

The efficacy of membrane transport differs with cell type even if the delivery vehicle and genetic material are the same. Our studies showed significant differences in the level of transfection of 22 kDa linear polyethyleneimine (PEI) treated plasmid DNA with luciferase gene in three breast cancer cell lines; MCF-7, SK-BR3 and T-47D [85]. Cellular targetability can be accomplished by cell surface receptors, such as hyaluronic acid (HA). Nanoparticles containing HA are selectively taken up by MDA-MB-435 cell line, which is rich in CD44 receptors [86]. There have also been many attempts to decorate the surface of nanoparticles with different kinds of targeting motifs to enhance cellular targetability by receptor-mediated endocytosis. For example, the RGD peptide sequence has been incorporated into nanoparticles to make use of the peptide’s ability to recognize integrins that are overexpressed on tumor cells or the angiogenic endothelial cells of the tumor vasculature [87]. Conjugation with polyethylene glycol (PEG) increases the serum half-life of nanoparticles, thereby increasing their pharmacokinetics and biodistribution [88]. Therefore, copolymers of PEG and polymeric nanocarriers containing the RGD peptide sequence are effective for cellular targeting and improved serum stability [89].

## 4. Synthetic Polymers for Gene Delivery

The cationic molecules which are currently under development as gene delivery vehicles are organic cations, including cationic lipids, polyamine-based polymers, chitosan-based polymers, dendrimers, and polyethyleneimine (PEI) [90,91,92]. These polymers interact with DNA and provoke the formation of compact nano-sized polyplexes [12]. The charge neutralized compact polyplex core protects enclosed nucleic acids from nucleases, and hence, maintain their stability and integrity until the cellular uptake process occurs [39,93,94]. Although PEI is a non-biodegradable polymer, it is included herein because it represents a polymer showing high transfection efficiency and could be made biodegradable by appropriate modifications.

### 4.1. Polyethyleneimine and Its Biodegradable Derivatives

**Figure 3:**
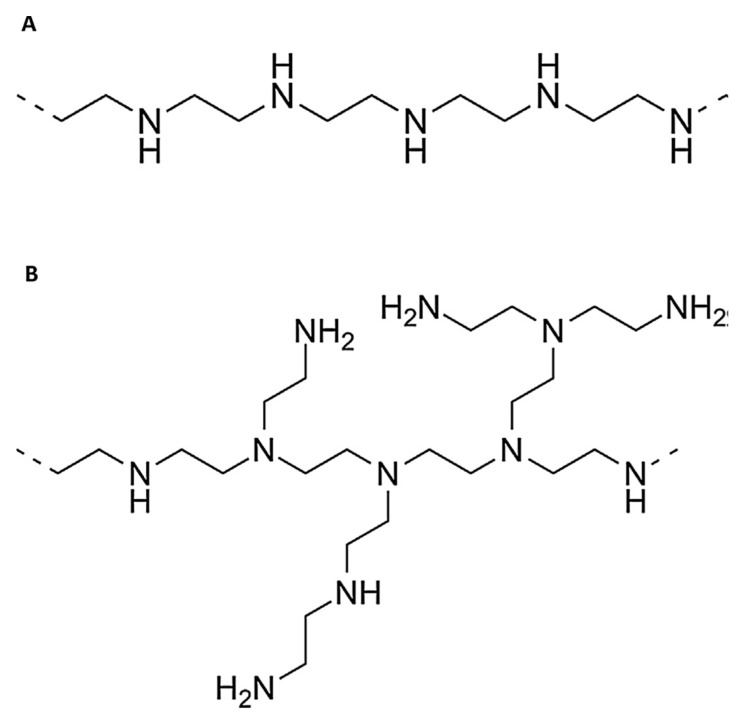
Chemical structures of linear (**A**) and branched (**B**) polyethyleneimine.

Amongst the various cationic polymers containing amine group in their backbone, polyethyleneimine has been the most extensively studied agent since 1995, when Boussif et al. described it as an effective gene transfection agent [95,96,97,98]. PEI has been synthesized in two different forms, branched and linear (Figure 3), and both forms have demonstrated high transfection capability both in vivo and in vitro [85,95,96,97,98,99]. The transfection efficiency of PEI depends on several factors, including, target cell type, molecular weight (MW) and structure of the PEI (branched as opposed to linear) [85]. Approximately 20% of the nitrogen of PEI is protonated under physiological conditions [100]. Therefore, PEI could envelope negatively charged DNA molecules, and protect them from lysosomal degradation and lead to higher expression of the transfected gene [101]. Using fluorescent-labeled PEI and DNA, Godbey et al. [102] and Venketeswaran et al. [85] found that PEI/DNA complexes attach to cell surfaces and migrate into clumps that are endocytosed. In addition, endocytosed PEI, whether administered with or without DNA, could undergo nuclear localization in the form of ordered structures. However, nuclear delivery of DNA was more facile with a 22 kDa linear PEI compared to that of branched PEI [85].

Despite its ability to deliver DNA and oligonucleotides in cellular and animal models, PEI suffers from the critical shortcoming of non-degradability that leads to severe cytotoxic effects [31,103,104]. In addition, stability in serum circulation, efficient intracellular release and low toxicity are important criteria for using cationic polymers as gene delivery vehicles. Therefore, several investigators have attempted to synthesize PEI derivatives with degradable linkages [104,105,106,107].

Lee et al. synthesized reducible linear PEI containing disulfides that exhibited transfection efficiency comparable to PEI and high cell viability [107]. Bifunctional cross-linkers, such as dimethyl-3,3′dithiopropionimidate (DTBP) and dithio-bis-succinimidyl propionate could react with primary amines, generating high molecular weight reducible polymers. PEI cross-linked with homobifunctional, amine reactive, reducible, and cross-linking reagents was tested for its transfection efficiency in CHO cells and showed that the modified polymers mediated different levels of transfection-based on cross-linking agent, the extent of cross-linking and N/P (nitrogen to phosphate) ratios [108]. An improvement in the efficiency of low-molecular-weight PEI is provided by the synthesis of dipicolylamine-based disulfide-containing zinc (II)-coordinated module (Zn-DDAC) [109]. Optimal Zn-coordinated polymeric vector induced up to 2-orders of magnitude higher luciferase activity than that of commercial transfection reagents. In a different approach, Albuquerque et al. constructed block co-polymers of PEI with poly(2-(dimethyl amino)ethyl methacrylate) (PDMA) and poly(2-(diethyl amino)ethyl methacrylate) (PDEA) for the optimization of DNA condensation and cellular transport [110].

**Figure 4:**
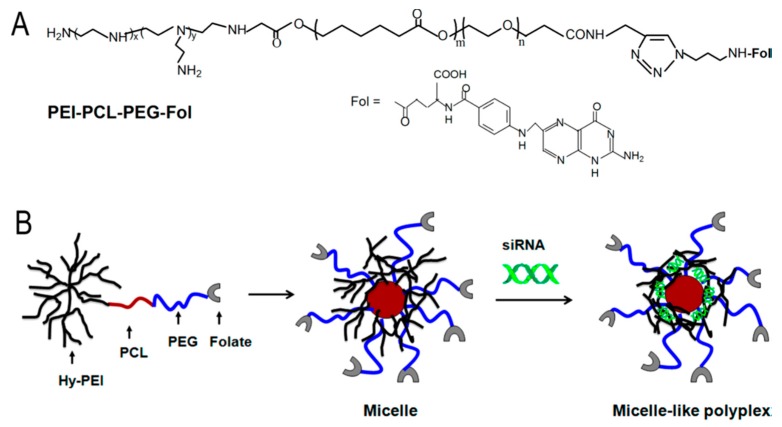
Chemical structure of polyethylenimine-graft-polycaprolactone-block-poly(ethylene glycol)-folate (PEI-PCL-PEG-Fol) (**A**) and schematic illustration of the micelle-like polyplex formation (**B**). Adapted with permission from Reference [114].

Heparin-polyethyleneimine (HPEI) nanogels are another group of biodegradable PEI conjugates [111]. After intravenous administration, HPEI degraded, and the degradation products were excreted through urine. HPEI nanogels had excellent transfection efficiency, low cytotoxicity, and better blood compatibility than 25 kDa PEI. In addition, pVSVMP/HPEI complexes inhibited the growth of pulmonary metastases [112]. Low MW PEI was also modified by the stepwise reactions with methylacrylate (aza-Michael reaction) and amidation with tetraethylenepentamine (TEPA) [113]. The resultant biodegradable copolymers were excellent transfection agents for plasmid DNA and siRNA in CHO and A549 cells and had target gene silencing ability without compromising biocompatibility. Targeted delivery of siRNA for gene silencing was accomplished by preparing polyethylenimine-graft-polycaprolactone-block-poly(ethylene glycol)-folate (PEI-PCL-PEG-Fol) (Figure 4) [114].

PEI-PCL-PEG-Fol/siRNA micelleplexes showed enhanced cellular uptake and in vitro gene silencing in SKOV-3 ovarian cancer cells that expressed a high level of folic acid receptor, compared to that of non-folate conjugated copolymers [114]. Similar results were found with xenograft models also. Miscelles prepared by grafting branched 25kDa, and PEG on poly[(ε-caprolactone)-co-glycolide] (CG) showed a high level of degradation after cellular internalization [115]. These micelle-based polyplexes showed high DNA transfection activity, as demonstrated by reporter gene-expression and siRNA mediated gene knockdown. PEI-related cytotoxicity was reduced by the incorporation of PEG, with the additional advantage of high serum stability of both DNA and siRNA polyplexes. Tumor targeting was also achieved by cross-liking low MW PEI with N-octyl-N-quaternary chitosan (OTMCS), and then conjugated with a trifunctional peptide (RGDC-TAT-NLS) [116]. The delivery vehicle, thus, prepared (OTMCS-PEI-R18) showed controlled degradation and DNA condensing and nuclear transportation capacity.

Ruan et al. modified low MW (1.2 kDa) PEI through a cross-linking reaction to introduce boric acid ester bond to make it susceptible to biodegradation by reactive oxygen species (ROS) [117]. Further modification of this polymer with substance P (SP) as the targeting ligand through PEG produced a conjugated polymer that had excellent biocompatibility, ROS cleavability ad transfection efficacy and gene silencing activity in vitro and satisfactory antitumor activity in vivo. A recent study shows that linking of 600 Da PEI with biodegradable bridges of aromatic rings could enhance the DNA binding ability of low MW PEI and increase its stability and transfection efficacy [118].

Grafting of PEI with chitosan, PEG and dextran also provided efficient and biodegradable gene delivery vehicles [119]. Conjugates of low MW PEI with depolymerized chitosans (7 and 10 kDa) interacted efficiently with DNA to produce nanoparticles of 100–160 nm [120]. These nanoparticles showed efficient transfection ability in vitro. Luciferase reporter gene analysis in male Balb/c mice receiving intravenous administration of the polyplex showed higher gene transfer ability compared to unconjugated chitosan and PEI-based polyplexes. Tseng et al. prepared PEI conjugates with dextran and folate and found that their toxicity was less than that of PEI [121]. The excellent transfection ability of these conjugates could be the release of plasmids from endosomes because the conjugated molecules hindered the protonation of PEI.

Another natural polymer used for modifying PEI is pullulan, a polysaccharide polymer consisting of maltotriose units [122]. PEI conjugated with different MWs of pullulan (5900 and 107,000 Da) was complexed with Apo B-siRNA, and injected into the liver of mice. Introduction of pullulan into PEI dramatically decreased mortality and lung damage in mice after systemic injection as compared to injection of PEI alone. The vector prepared with high MW pullulan was more efficient in serum stability and gene expression compared to that prepared with the low MW pullulan. Wang et al. synthesized pullulan-PEI (P-PEI) conjugates and modified it by conjugating with folic acid. The resulting polymer was biodegradable, and transported pDNA with excellent efficacy in different cell types [123]. The P-PEI-FA/pDNA complex showed higher gene transfection and gene silencing efficiency at N/P ratio of 6.25 compared to the vector lacking pullulan. P-PEI-FA/siRNA can also deliver FAM-labeled siRNA to endosomes and escape. An amphiphilic bifunctional derivative of pullulan was also synthesized by conjugating desoxycholic acid and branched 1000 Da PEI onto pullulan [124]. The resulting polymer conjugate showed excellent blood compatibility, low cytotoxicity and sustained drug release profile and good DNA-binding ability. These micelles could efficiently transport the p53 gene into MCF-7 cells, and the expressed exogenous p53 protein inhibited the growth of these cancer cells.

Certain amino acids, including lysine-histidine peptides and arginine-rich peptides, were also used to modify low MW PEI to transport plasmids and siRNA [125,126]. The conjugates prepared by linking cell penetrating protein (CPP) with PEG showed excellent gene transfection efficiency in two different lung cancer cell lines, with luciferase reporter gene expression in mouse lungs [127]. The conjugates sizes were generally <300 nm, thus, enabling them to penetrate through the mucus lining of the lung and reach the target cells. Taken together, there are multiple approaches to reduce the toxicity of PEI by conjugation with biodegradable molecules.

### 4.2. Poly-β-Aminoesters

**Figure 5:**
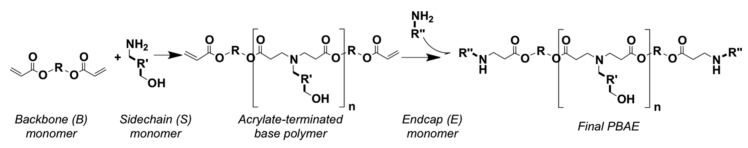
PBAE synthesis reaction scheme. Adapted with permission from Reference [129].

Aminoesters are a group of biodegradable polymers that have cationic amino groups and hydrolysable ester linkages [128]. A typical synthesis of a poly-β-aminoester (PBAE) is illustrated in Figure 5.

PBAE molecules are capable of interacting with DNA by electrostatic forces to produce nanoparticles of 100–200 nm diameter [130]. They are non-cytotoxic and biodegradable with a half-life of 1–7 h in aqueous solutions [131,132]. Guerrero-Ca’zares et al. synthesized a panel of PBAE and tested their efficacy in human glioblastoma cells in vitro and in vivo. Nanoparticles formed with PBAE and DNA showed excellent transfection ability in brain tumor initiating cells in vitro even when these cells were grown in 3D oncospheres. In addition, cell specificity was evident in the patient-derived orthotopic murine model of human glioblastoma [131].

**Figure 6:**
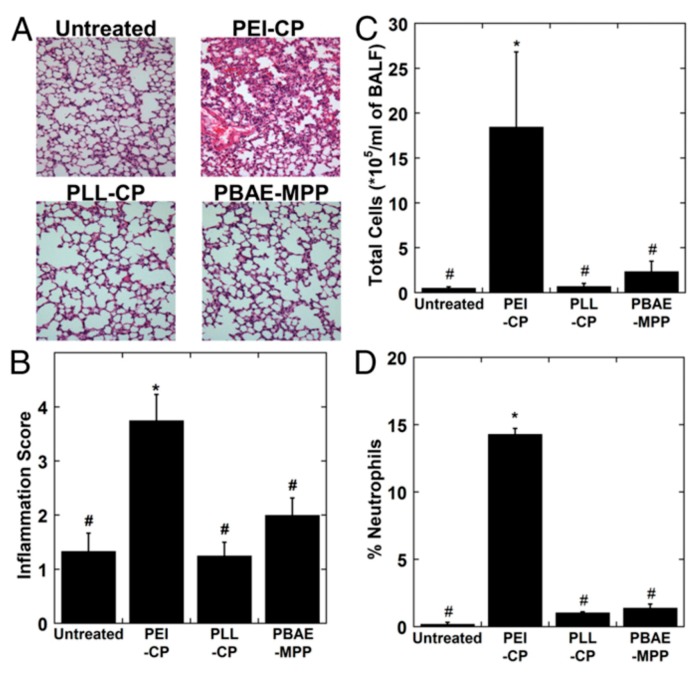
In vivo safety profile of DNA-NPs. (**A**) Representative images of lung parenchyma 24 h after administration of DNA-NPs. (**B**) Histopathological scoring of lung inflammation. (C and D) Total cell counts (**C**) and % neutrophils (**D**) in bronchoalveolar lavage fluid following a single administration of DNA-NPs. Data represent the mean ± SD (n = 3–5). The differences are statistically significant (*p* < 0.05) compared with untreated control (*) or mice dosed with Conventional PEI nanoparticles (PEI-CPs) (#). Adapted with permission from Reference [135].

Enzyme-catalyzed copolymerizaton of the lactone with dialkyl diester and amino diols produced polyamine(co-esters) that could condense DNA and undergo facile cellular transport in a variety of cells, including human embryonic kidney 293, U87-MG, and 9L cell lines [133]. Targeted delivery of the pro-apoptotic TRAL gene by these agents showed significant inhibition of tumor growth in tumor xenograft models, with minimal toxicity in vitro and in vivo. PBAE could also recognize minicircle DNA to form nanoparticles for delivery in kidney 293 cells and mouse embryonic fibroblasts, as model cell types [134]. Intraperitoneal injection of minicircle DNA in vivo resulted in high transgene expression, and the level of expression was double with PBAE complexed DNA compared to control experiments. Mastorakos et al. further developed highly stable PBAE-based DNA nanoparticles and tested their ability to penetrate the nanoporous and highly adhesive mucous airways as a potential therapeutic approach for respiratory diseases and lung cancer [135]. In addition, these PBAE-based mucus-penetrating DNA nanoparticles (PBAE-MPPs) provided uniform and high-level transgene expression throughout the mouse lungs, superior to several gold-standard gene delivery systems. Transgene expression was robust for more than four months by a single administration in mice. The safety profile of these PBAEs was excellent, following intratracheal administration (Figure 6). In addition, surface-modified poly(lactic-co-glycolic acid) (PLGA)/poly-(β-aminoester) (PBAE) nanoparticles (NPs) have shown great promise in pulmonary gene delivering and genes editing [136].

PBAE conjugation with 5 kDa PEG was used to increase the circulation half-life of PBAE complexed DNA nanoparticles [136]. The PEG-coated nanoparticles penetrated healthy brain parenchyma and orthotopic brain tumor tissues in rats and achieved widespread transgene expression throughout the tumors in vivo. In addition, these brain penetrating nanoparticles loaded with an anti-cancer plasmid DNA improved the survival time in two aggressive orthotopic brain tumor models in rats [137].

Kauffman et al. synthesized a family of low toxicity poly(amine-co-ester) (PACE) terpolymers via an enzyme-catalyzed polymerization for potential use in transporting plasmid DNA, miRNA, and siRNA [138]. The advantage of PACE polymers is that they could be synthesized in a fine-tuned manner, based on cell type and nucleic acid characteristics. Another approach for cell-type specific delivery is the synthesis of Branched poly(Ester Amine) Quadpolymers (BEAQs) via the Michael addition reactions from small molecule acrylate and amine monomers and then end-capping with amine-containing small molecules to assess the influence of polymer branching structure on transfection [139]. BEAQs with moderate degrees of branching were optimal for delivery in serum-containing media. Reducible branched ester-amine quadpolymers (rBEAQs) are also under development to co-encapsulate and deliver DNA plasmids and RNA oligos for applications, such as non-viral CRISPR-mediated gene editing, utilizing Cas9 DNA and siRNA codelivery [140].

### 4.3. Poly-L-Lysine (PLL)

**Figure 7:**
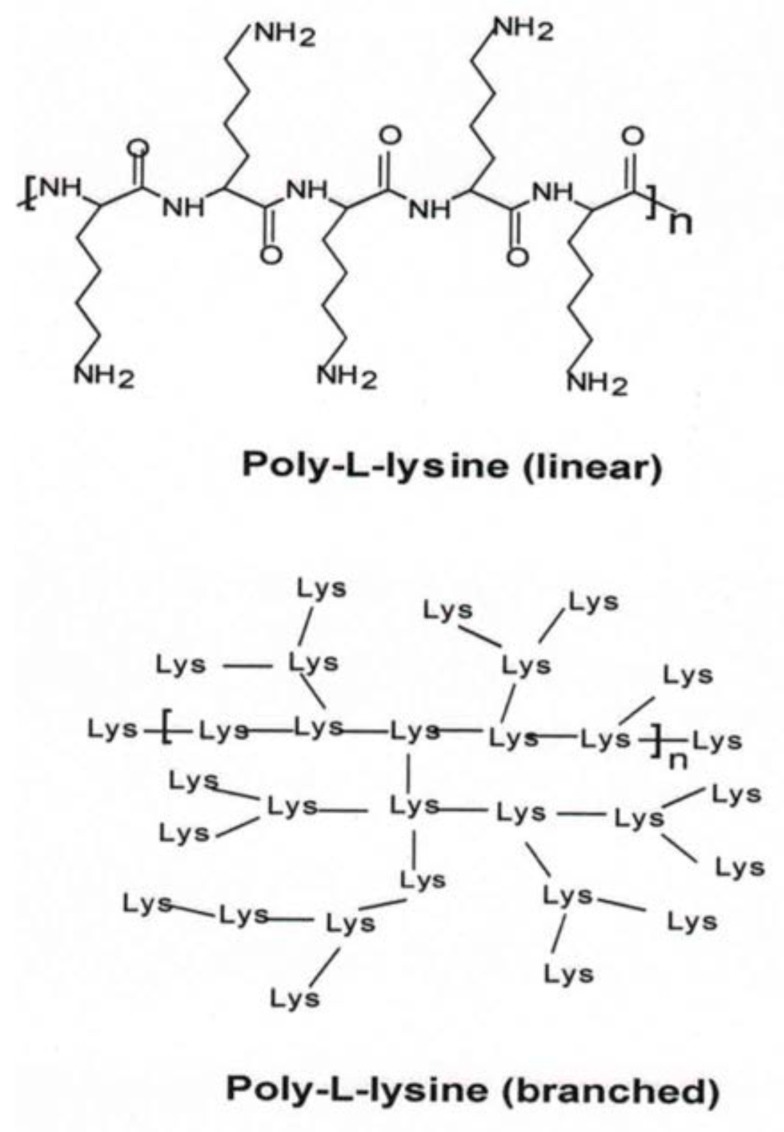
Chemical structures of linear and branched poly-L-lysine.

The cationic peptide, PLL (Figure 7) and its low molecular weight analogues, oligolysines were studied for their ability to collapse DNA to nanoparticles and transport DNA to cells [58,141].

Studies by Nayvelt et al. provided mechanistic insights into oligo- and poly-L-lysine-mediated DNA collapse to nanoparticles, with typical morphologies of toroids, spheroids, cubes and rods [58]. Korolev et al. found salt-dependent and salt-independent regimes in the interaction of oligolysines with plasmid DNA [142]. Modification of PEI with PLL enhanced the transfection efficacy of PEI in HeLa cells, in addition to significantly reducing its toxicity [143]. In a recent report, Malik et al. showed that polylysine-modified PEI could provoke genetically engineered mesenchymal stem cells for combinational suicidal gene therapy in glioblastoma [144]. Kodama et al. developed dendrigraft PLL (DGL) as an alternative to PLL and complexed it with γ-polyglutamic acid (γ-PGA) for gene delivery [145]. The ternary complex formed with DNA showed high transfection efficiency in the liver, lungs and spleen of experimental animals. Other investigators are synthesizing copolymers of poly-L-lysine for enhanced gene delivery applications [146].

Several other cationic peptides have also found use as biocompatible and biodegradable gene delivery vehicles. Cell penetrating proteins (CPPs) are a group of cationic peptides that can condense DNA and facilitate DNA/siRNA delivery [147]. CPPs contain <30 amino acids and their design is inspired by a trans-activating transcriptional activator (Tat) of human immunodeficiency virus 1 (HIV-1) with the amino acid sequence of GRKKRRQRRRPQ and penetrating peptide of the sequence, RQIKIWFQNRRMKWKK [148]. DNA transporting efficacy of CPPs depends on their sequence length and on the position of the arginine residue in the peptide sequence. Linear and flexible versions of CPPs have been synthesized [147]. Tumor targeting peptides, including RGD and Lyp-1, have also been developed to bind to epithelial cells to transport the DNA cargo directly to tumor cells [149].

## 5. Natural Carbohydrate Polymers for Gene Delivery

**Figure 8:**
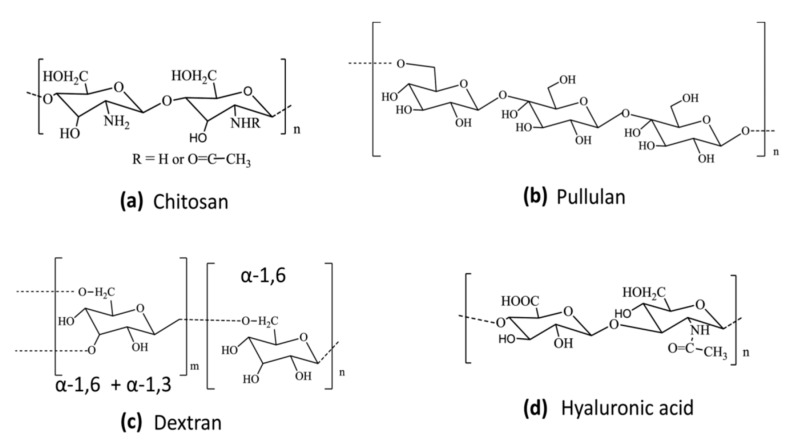
Chemical structures of commonly used natural polymers for gene delivery. Chitosan (**a**) is a linear amino polysaccharide comprising randomly distributed β(1,4)-linked D-glucosamine and N-acetyl-D-glucosamine units. Pullulan (**b**) is a water-soluble linear polysaccharide, with α-1,4-glucopyranose and-1,6-glucopyranose units. Dextran (**c**) is a carbohydrate polymer composed predominantly of α-1,6-linked glucopyranose units with a low degree of 1,3-branching. Hyaluronic acid (HA) (**d**) is an anionic polysaccharide, a glycosaminoglycan (GAG) composed of repeating polymeric disaccharides of D-glucuronic acid and N-acetyl-D-glucosamine linked by a glucuronidic β (1→3) bond.

Several natural polymers (Figure 8) have been studied for gene delivery as these agents are relatively non-toxic and biodegradable [12,150].

### 5.1. Chitosan

**Figure 9:**
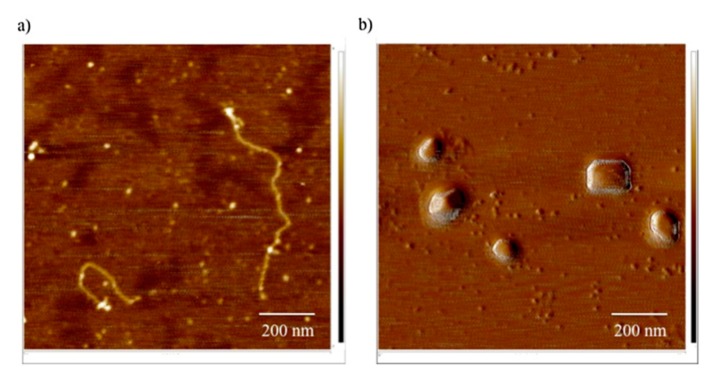
AFM images of DNA and DNA/chitosan complexes. (**a**) Single chains of DNA prepared in water at a concentration of 7.5 x 10^-3^ g/L and (**b**) chitosan-DNA complexes formed at a charge ratio of 0.41 with 50 kDa chitosan in 0.01 M NaCl. Scale bar is 200 nm. Adapted with permission from Reference [154].

Among the natural carbohydrate polymers, chitosan (Figure 8a) has received the most attention as a nanoparticulate drug and gene delivery vehicle [151]. Chitosan condenses DNA to nanoparticles at acidic and neutral pH, due to the presence of amino groups that confer a high positive charge density [152]. It is obtained by the N-deacetylation of chitin, and has randomly distributed β(1,4)-linked D-glucosamine and N-acetyl-D-glucosamine units. Low immunogenicity, biocompatibility and minimal cytotoxicity are some of the advantages of using chitosan as a gene delivery vehicle. Hydrogels formed by it are compatible with biodegradation by lysozyme and chitosanase enzymes and have low toxicity [153]. As described in the previous section, this property of chitosan has been utilized in preparing conjugates with other polymers, such as PEI, to render them biodegradable. The interaction between chitosan and DNA is electrostatic in origin, as reported for the majority of polycations [154]. Since chitosan is a weak base with a pK_a_ of 6.5, pH is an important factor governing facile interaction of its amino groups with DNA. Compaction of DNA with low MW chitosan gives nanoparticles with an average radius of ~150 nm diameter (Figure 9). Such an interaction appears to be strong enough that the chitosan-DNA complex does not dissociate until it has entered the cell. Once its role is over, chitosan is degraded into the common amino-sugar, N-acetyl glucosamine, which is incorporated into the metabolic pathway of glucoproteins, and is subsequently excreted form the body [155].

The transfection efficiency of chitosan depended on the degree of deacetylation and MW of the chitosan, pH, the presence of serum, chitosan to DNA charge ratio and cell type. The poor solubility in water, low specificity and low transfection efficiency have been major hindrances to its development as an effective gene delivery vector [156]. Morris et al. prepared chitosans of a wide range of MW and degree of deacetylation to determine the optimum conditions of this biopolymer for gene delivery applications [157]. Chitosans of medium MW (49–51 kDa) and a high degree of deacetylation produced stable, uniform-sized nanoparticles. Biological studies with the spherical nano-sized polyplexes formed between 50 kDa chitosan of 94% degree of deacetylation and pEGFP plasmid DNA (N/P ratio = 5) showed excellent gene transfection efficiency at pH 6.5 in HeLa cells. This complex had no cytotoxicity, indicating its potential use as a gene delivery vehicle. This study also showed that chitosan in the MW range of 49–51 kDa could be useful in condensing DNA to deliver nanoparticles of near-uniform size (~50 nm) for gene delivery applications [157].

The presence of hydroxyl and amino groups in the backbone of chitosan makes it amenable to the chemical modification to improve its chemical properties and effectiveness. Quaternization of the amino groups could be used to render medium and low MW chitosans water-soluble over a wide range of pH and to confer controlled cationic character [157]. Cellular targetability could be introduced by conjugation of folic acid through the quarternized derivative because of the presence of folate receptors on tumor cell [157,158]. The conjugated polymer could provoke plasmid DNA condensation to uniformly-sized nanoparticles of ~140 nm size and high positive surface charge density. The pH profiles of folic acid conjugated trimethylated depolymerized chitosan suggested that the polymers had endosomal disruption capacity, and the gel electrophoretic mobility band retardation showed efficient condensation of DNA. Folic acid derivatized chitosan, and its DNA complex were less toxic and hemocompatible than that of PEI and its DNA complex. The chitosan complex also showed excellent transfection efficiency, as tested in human KB epidermoid cell line. Plasmid pGL3 was transported to the cell nucleus [159]. The water-solubility and transfection efficiency of chitosans could be improved when the depolymerized trimethylated chitosans were modified with the histidine moiety [159]. Spherical nanoparticles could be formed, and these derivatives could buffer in the pH range of 10 to 4. The transfection efficiency of this chitosan conjugate was comparable to that of control PEI. Taken together, the enhanced cellular and nuclear uptake of chitosan conjugates show that chitosan can be modified to yield highly efficient gene delivery vehicles.

Another group of investigators synthesized thiolated methylated N-(4-N,N-dimethyl- aminobenzyl N,O-carboxymethyl chitosan derivatives to improve the solubility and delivery properties of chitosan [160]. These derivatives had a higher solubility in water compared to that of chitosan and had no significant toxicity against Hek293 kidney cell line in comparison to that of chitosan. Rahmani et al. prepared trimethyl chitosan, methylated 4-N,Ndimethyl aminobenzyl N,O-carboxymethyl chitosan and thiolated trimethyl aminobenzyl chitosan and showed that these polymers could condense DNA to nanoparticles [161]. These nanoparticles exhibited facile transfection in SKOV-2 ovarian and MCF-7 breast cancer cell lines. With siRNA, O-methyl-free N,N,N-trimethylated chitosan showed excellent gene silencing activity comparable to that of PEI in H1299 human lung cancer cells expressing firefly luciferase, indicating the use of chitosan derivatives in siRNA delivery [162]. A recent report showed that folic acid-modified polyethylene glycol-chitosan oligosaccharide lactate nanoparticles facilitated siRNA delivery targeted to multiple genes in a pancreatic cancer xenograft model and strongly inhibited retroperitoneal invasion and inhibited peritoneal dissemination compared to the other nanoparticles [163]. Several other modified forms of chitosan are under active investigation for gene delivery applications of different cell types [164,165,166,167].

### 5.2. Pullulan

Pullulan (Figure 8b) is a water-soluble linear polysaccharide, with α-1,4-glucopyranose and α-1,6-glucopyranose units [168,169]. It is non-toxic, non-immunogenic, non-carcinogenic and non-mutagenic and has found applications in food packaging and pharmaceutical industries.

Derivatization of pullulan with cationic molecules, such as spermine, can produce positively charged nanoparticles with an excellent binding affinity toward DNA and gene transfection efficacy [170]. Priya et al. synthesized cationic pullulan by conjugating it with protamine [171]. This polymer could protect DNA from degradation and had excellent haemocompatibility and improved cellular viability. A recent study showed the synthesis of a redox-responsive system by combining a charge- reversible pullulan derivative (CAPL) and disulfide-containing PBAE for the co-delivery of a gene and a chemotherapeutic agent [172]. This agent could condense DNA and deliver a dye-labeled pDNA in human hepatoma HepG2 cells. Pullulan conjugation also enhanced the gene delivery efficacy of PAMAM dendrimer in HepG2 cell line [173].

### 5.3. Dextran

Dextran (Figure 8c) is another carbohydrate polymer with applications in biomedicine and gene delivery. It is composed predominantly of α-1,6-linked glucopyranose units with a low degree of 1,3-branching [12,174]. Cationic, biodegradable dextran hydrogel nanoparticles could be prepared by derivatization with cationic methacrylate monomers for siRNA delivery [175]. In addition, negatively charged dextran sulfate could form polyelectrolyte complexes with positively charged polymers, such as poly-L-arginine for siRNA delivery [176]. Dextran-grafted branched PEI was found to be effective to improve the stability of the PEI complexes with DNA in the presence of BSA [177]. Dextran-PEI conjugates were less toxic than unmodified PEI, as determined by the MTT assay [178]. Nanoparticles formed by the complexation of polyallyamine (PAA)-dextran conjugate with DNA were more efficiently transfected than that of PAA-DNA nanoparticles [179]. Chitosan-dextran conjugates showed excellent transfection efficiency of frizzled-related protein 4 (SFRP4) in both JU77 and ONE58 cell lines [180]. Histidine (H)-containing peptide-grafted dextran (D-RxHy) displayed a 6-8-fold higher luciferase expression compared to that of 25 kDa branched PEI [181].

### 5.4. Hyaluronic Acid (HA)

HA (Figure 8d) is an anionic polysaccharide that has found biomedical applications, including drug and gene delivery. It is composed of D-glucuronic acid and N-acetyl-D-glucosamine [182]. It binds with the CD44 receptor, that is overexpressed on the surface of many types of tumor cells, and hence, it is an excellent vehicle for targeted delivery of genes and drugs to cancer cells. HA can form nanogels by electrostatic interaction with polycations, such as polyarginine [183]. The interaction of siRNA and HA by van der Waals forces has been exploited for gene silencing in a CD44-positive human osteocarcinoma cell line (MG63) and in human mesenchymal stromal cells [184]. Nanoparticle formulations prepared by the complexation of HA conjugated PEG (HA-PEG) and HA-PEI produced excellent results in gene transfection and gene expression with negligible cytotoxicity in HeLa and A549 human lung cancer cell lines [185]. HA was complexed with PEI and the complex used to deliver MMP13 gene in a mouse model of liver fibrosis, with excellent results [186]. Lipoplexes containing plasmid DNA within polyelectrolyte multilayers composed of glycol-chitosan (Glyc-CHI) and hyaluronic acid (HA) was used to transfect NIH2T3 fibroblasts and HEK293 kidney cells in vitro [187]. Ternary nanocomplexes of HA conjugates with poly(hexamethylene biguanide) and chitosan were to deliver an anti-KRAS siRNA to colorectal cancer cells, exploiting the interaction of HA with CD44 as a means to achieve selective targeting of CD44-positive cancer cells [188]. Multilayers of HA/PEI were also constructed to deliver siRNA and gene silencing [189].

We have summarized work on four natural biodegradable polymers above; however, there are several other molecules, such as heparin, chondroitin sulfate, and alginate that are under investigation as gene delivery vehicles [12].

Table 1 shows a summary of the modifications on polymers to confer biodegradability and enhance cellular transportation.

## 6. Concluding Remarks

Polymer-based non-viral gene carriers have been developed, due to their merits in safety, including the avoidance of potential immunogenicity and toxicity, the possibility of repeated administration, and the ease of establishing good manufacturing practice (GMP) [50]. Although preclinical studies and human clinical trials demonstrated therapeutic benefit of several gene therapy approaches, efficient gene delivery remains a key obstacle for moving several drug candidates to the clinic.

There are several systemic and cellular barriers, including serum proteins in the bloodstream, cell membrane, endosomal compartment and nuclear membrane. Polymer design and modifications have been successfully used to circumvent these barriers. Structural modifications include incorporations of guanidinium group, carboxyl group, disulfide bond, alkyl chain, branching, acetyl groups, benzoyl groups, and quaternary nicotinamide moieties to facilitate DNA condensation, cellular uptake, endosomal escape, nuclear entry and gene expression (Table 1). Polymer complexed DNA nanoparticles can be administered by injection, infusion and/or inhalation for realizing the potential therapeutic benefits.

## 7. Future Directions

The cellular transport of DNA is a complicated process, and the mechanistic aspects of this process are not clearly understood at present. The development of polymeric gene delivery vehicles focused on molecules that could compact DNA to nanoparticles and transport the genetic material in a facile manner, produce no immunogenic response and degrade to small molecules that could easily get eliminated from the body. Although significant progress has been made in the development of DNA condensation agents, the discovery of an ideal biodegradable delivery vehicle has not been accomplished. Modification of currently existing natural and synthetic polymers with linker groups that can undergo hydrolysis and enzymatic degradation is making significant progress.

## Figures and Tables

**Table 1:** Polymer modifications to facilitate biodegradability and/or cellular transport.

Polymer	Modification	References
Polyethleneimine (PEI)	Disulfide linkage PDMA/PDEA copolymer Heparin TEPA PCL-PEG-FA PCL-CG Chitosan Boric acid Aromatic ring bridges Dextran Pullulan/FA Desoxychlic acid Amino acids	[107,108,109] [110] [111,112] [113] [114] [115] [116,119,120] [117] [118] [121] [122] [123,124] [125,126,127]
PBAE	PLGA/PEG PACE BEAQ	[136] [138] [139]
PLL	PEI γ-PGA	[143,144] [145]
Chitosan	FA Dimethylaminobenzyl FA/PEG	[157,158] [160,161] [163]
Pullulan	Spermine Protamine PBAE PMAM	[170] [171] [172] [173]
Dextran	PEI/Polyarginine PAA Chitosan Histidine	[176] [179] [180] [181]
Hyaluronic Acid, HA	PA/PEI PEG Glycol Biguanidine	[183,186] [185] [187] [188]
